# Gamma-aminobutyric acid (GABA) improves salinity stress tolerance in soybean seedlings by modulating their mineral nutrition, osmolyte contents, and ascorbate-glutathione cycle

**DOI:** 10.1186/s12870-024-05023-6

**Published:** 2024-05-06

**Authors:** Zhao Qian, Liu Lu, Wei Zihan, Bai Qianyue, Zhao Chungang, Zhang Shuheng, Pan Jiali, Yu Jiaxin, Zhang Shuang, Wei Jian

**Affiliations:** 1 School of Life Sciences, Changchun Normal University, Changchun, 130032 China; 2 School of Agriculture, Jilin Agricultural University, Changchun, Jilin 130118 China; 3 School of Life Sciences, Wuhan University, Wuhan, 430072 China

**Keywords:** Climate change, Abiotic stress, Salinity, Physiology, Antioxidant mechanism, Ion homeostasis

## Abstract

**Background:**

In plants, GABA plays a critical role in regulating salinity stress tolerance. However, the response of soybean seedlings (*Glycine max* L.) to exogenous gamma-aminobutyric acid (GABA) under saline stress conditions has not been fully elucidated.

**Results:**

This study investigated the effects of exogenous GABA (2 mM) on plant biomass and the physiological mechanism through which soybean plants are affected by saline stress conditions (0, 40, and 80 mM of NaCl and Na_2_SO_4_ at a 1:1 molar ratio). We noticed that increased salinity stress negatively impacted the growth and metabolism of soybean seedlings, compared to control. The root-stem-leaf biomass (27- and 33%, 20- and 58%, and 25- and 59% under 40- and 80 mM stress, respectively]) and the concentration of chlorophyll a and chlorophyll b significantly decreased. Moreover, the carotenoid content increased significantly (by 35%) following treatment with 40 mM stress. The results exhibited significant increase in the concentration of hydrogen peroxide (H_2_O_2_), malondialdehyde (MDA), dehydroascorbic acid (DHA) oxidized glutathione (GSSG), Na^+^, and Cl^−^ under 40- and 80 mM stress levels, respectively. However, the concentration of mineral nutrients, soluble proteins, and soluble sugars reduced significantly under both salinity stress levels. In contrast, the proline and glycine betaine concentrations increased compared with those in the control group. Moreover, the enzymatic activities of ascorbate peroxidase, monodehydroascorbate reductase, glutathione reductase, and glutathione peroxidase decreased significantly, while those of superoxide dismutase, catalase, peroxidase, and dehydroascorbate reductase increased following saline stress, indicating the overall sensitivity of the ascorbate-glutathione cycle (AsA-GSH). However, exogenous GABA decreased Na^+^, Cl^−^, H_2_O_2_, and MDA concentration but enhanced photosynthetic pigments, mineral nutrients (K^+^, K^+^/Na^+^ ratio, Zn^2+^, Fe^2+^, Mg^2+^, and Ca^2+^); osmolytes (proline, glycine betaine, soluble sugar, and soluble protein); enzymatic antioxidant activities; and AsA-GSH pools, thus reducing salinity-associated stress damage and resulting in improved growth and biomass. The positive impact of exogenously applied GABA on soybean plants could be attributed to its ability to improve their physiological stress response mechanisms and reduce harmful substances.

**Conclusion:**

Applying GABA to soybean plants could be an effective strategy for mitigating salinity stress. In the future, molecular studies may contribute to a better understanding of the mechanisms by which GABA regulates salt tolerance in soybeans.

**Supplementary Information:**

The online version contains supplementary material available at 10.1186/s12870-024-05023-6.

## Introduction

The salinity of soils is one of the most important abiotic stresses that negatively impact agricultural productivity globally. It is estimated that 3600 million hectares (Mha) of arable land are lost to the salinization of soil out of 5200 Mha, resulting in a loss of USD 27.5 billion each year [1]. Climate change and inefficient agricultural practices are expected to increase the salinity of soils, making them unsuitable for agricultural use. Moreover, the world population is expected to reach 10 billion by 2050, which will increase the demand for food production by 70%. Further pressure will be exerted on the declining area of arable land [2]. Therefore, rapid salinization negatively affects both ecological and socioeconomic values [3]. The excessive accumulation of toxic salt ions, such as Na^+^ and Cl^−^, in plants causes osmotic stress, ionic toxicity, and nutritional deficiencies [4, 5]. Furthermore, soil salinity results in a decrease in photosynthetic abilities, reduced nutrient uptake, destabilization of membranes, impairment of antioxidant defense mechanisms, disrupted metabolism, and leakage of cellular membranes [4, 6]. Plants have developed several mechanisms to protect themselves against salinity-induced damage. Among these are the (i) production of osmolytes, (ii) removal of the toxic salt ion Na^+^ and Cl^−^ or their compartmentalization into vacuoles, and (iii) upregulation of antioxidant mechanisms to eliminate excessive reactive oxygen species (ROS) [5, 7–9].

In plants, reactive oxygen species (ROS) are known to play a significant role in signaling and stress response. The common ROS in plants includes superoxide anion (O_2_^•−^), hydroxyl radicals (•OH), and hydrogen peroxide (H_2_O_2_). When their accumulation exceeds the capacity of the plant’s antioxidant defense mechanisms, they can cause oxidative stress, resulting in protein, lipid, and DNA degradation [7]. Plants have evolved intricate antioxidant defense systems to combat the detrimental impacts of ROS, which include both antioxidant enzymes and antioxidant compounds [4]. The enzymatic antioxidant defense system consists of several enzymes such as superoxide dismutase (SOD), catalase (CAT), peroxidase (POD), and glutathione peroxidase (GPX). The enzyme SOD converts the O_2_^•−^ into H_2_O_2_. Subsequently, it is metabolized by the actions of POD, CAT, and GPX enzymes. Hence, they play vital roles in antioxidant mechanisms to break down H_2_O_2_ into water and oxygen or by utilizing it as a substrate to detoxify various organic and inorganic compounds, thereby protecting cells from stress-induced oxidative damage [9–11]. Additionally, the ascorbate-glutathione (AsA-GSH) cycle further eliminates excessive H_2_O_2_ through the coordinated actions of enzymes, including ascorbate peroxidase (APX), glutathione reductase (GR), and monodehydroascorbate reductase (MDHAR) and dehydroascorbate reductase (DHAR). This cycle performs protective roles for cells by eliminating excessive H_2_O_2_ and maintaining cellular redox balance [12, 13]. Hence, plants possessing robust antioxidant defense mechanisms are deemed more resilient to salinity stress [12, 14].

Moreover, plants also utilize osmolytes (proline, glycine betaine, soluble sugar, amino acids) to cope with osmotic stress, including salinity [15, 16]. They play multiple roles in coping with salinity stress. These include maintaining osmotic balance, safeguarding photosynthetic pigments, stabilizing proteins, scavenging ROS, preventing ionic toxicity, and regulating cell division and gene expressions [9, 15–17].

The uptake and homeostasis of ions are essential for the normal growth of plants. It is important to recognize that salt stress can cause ion toxicity and can inhibit the absorption of essential mineral ions such as Mg^2+^, Mn^2+^, Zn^2+^, B^3+^, K^+,^ and Fe^2+^ [18, 19], damaging physiological conditions and growth. Several studies have investigated how salt stress affects specific types of ions [20–22]. As the ionic balance in plants is intricate and integral, ionomics must be used to investigate the mechanisms responsible for salt tolerance in plants.

Extensive research has been conducted on increasing crop tolerance to abiotic stress through breeding programs. According to recent studies, the application of exogenous factors such as proline, polyamines, melatonin, naphthalene acetic acid, and Gamma aminobutyric acid (GABA) has proven to be an effective method for improving plant tolerance to salt stress and thereby increasing crop yield [9, 23–26]. GABA is a nonprotein amino acid synthesized in mitochondria through the GABA shunt. In plants, GABA serves as both a metabolite and a signaling molecule, actively participating in various physiological processes, particularly under stressful conditions [27, 28]. This multifunctionality enables plants to develop tolerance to salinity. As reported by previous studies, external GABA application mitigates salinity injury by modulating antioxidant enzymes to maintain low ROS concentration, regulating nitrogen metabolism, and osmolyte production, resulting in greater tolerance to salinity stress in various crops [24, 29–32].

Cultivated soybeans (*Glycine max* L.) hold significant economic importance globally as a crucial crop species. Over recent decades, the demand for soybean cultivation has shown a consistent rise. Reports indicate that this oil-seed legume crop contributes to approximately 80% of the total global legume productivity [33]. However, cultivated soybean plants generally exhibit higher sensitivity to salt compared to their wild counterparts [34]. Given the reduced productivity observed under salinity stress, enhancing the salt tolerance of this crop becomes imperative to sustain its productivity in soils affected by salinity.

Although the effects of salinity on soybeans have been extensively studied, the effects of GABA have received little attention. Moreover, the knowledge of GABA interactions during saline stress and how GABA modulates physiological and biochemical changes under saline stress in commercially critical cereal crop species, such as soybeans, remains elusive. Based on our hypothesis, saline stress is expected to hinder the growth of soybean plants. However, we anticipate that exogenous GABA application may mitigate the morphophysiological and biochemical damage induced by saline stress. To test our hypothesis, the impact of GABA (2 mM) application was investigated on growth, ion homeostasis, ROS accumulation, osmolyte accumulation, and antioxidant mechanisms in soybean plants subjected to saline stress (0, 40, and 80 mM NaCl and Na_2_SO_4_ at a 1:1 molar ratio). Therefore, we aimed to unravel the impact of exogenous GABA on soybean growth and physio-biochemical processes by considering the changes in (i) mineral ion homeostasis through the reduction of Na^+^ and Cl^−^ uptake; (ii) growth, photosynthetic pigment, and osmolyte production; and (iii) enzymatic and nonenzymatic defense mechanisms to reduce saline stress-induced oxidative damage.

## Materials and methods

### Study area and experimental conditions

The study was conducted at the College of Agriculture Sciences. The soybean seeds were sterilized with magnesium chloride (MgCl_2_, 0.1%) solution for 5 min, followed by five washes with distilled water. The seeds were then planted in plastic pots (15 cm in diameter) filled with 3 kg of soil, and a hole was placed at the bottom for drainage. The seedlings were initially watered with tap water every three days using a weighing method. The pots were regularly watered and weighed to replenish lost water due to evaporation and transpiration. The soil relative water content (SRWC) was determined using the following formula:

1 $$\mathrm{SRWC}\;=\;(\lbrack({\mathrm W}_{\mathrm{soil}}\;-\;{\mathrm W}_{\mathrm{pot}}\;-\;{\mathrm{DW}}_{\mathrm{soil}})/\;({\mathrm W}_{\mathrm{FC}}\;-\;{\mathrm W}_{\mathrm{pot}}\;-\;{\mathrm{DW}}_{\mathrm{soil}})\rbrack\;\ast\;100)$$

In this situation, W_soil_ is the weight of soil (pot + soil + water); W_pot_ is the weight of an empty pot; and DW_soil_ is the weight of dry soil, whereas W_FC_ is the soil weight at field capacity.

Two weeks after sowing, 36 pots containing uniform seedlings (*n* = 1) were selected and divided into six groups for the application of saline stress (NaCl and Na_2_SO_4_ at a 1:1 molar ratio) and GABA treatment. The three groups were treated with saline stress at different concentrations, namely, 0 mM, 40 mM, and 80 mM. The plants in the three remaining groups were also given saline stress but were sprayed with GABA solution (100 ml of 2 mM each at 15, 22, and 30 days after sowing). In an initial experiment, plants were grown with various GABA concentrations (0.5 mM to 2 mM) under 40 mM salinity stress (Supplementary Table 1). Moreover, the optimal GABA concentration was determined by measuring the improvement in soybean seedling growth under 40 mM saline stress. Finally, 7-week-old soybean plants were harvested to evaluate growth and physiological characteristics. For further laboratory testing, the samples harvested for physiological analysis were immediately frozen in liquid nitrogen and stored at − 80 °C.

### Measurement of growth indices

The shoot height and root length were measured using a manual. After the plants were separated into leaves, stems, and roots, their fresh weights were measured using an electric balance. In the next step, the leaves stems, and roots were oven-dried at 105 °C for 30 min, followed by drying at 75 °C until a constant weight was achieved. An electric balance was subsequently used to determine the dry weight of the plants.

### Determination of chlorophyll a, chlorophyll b, and carotenoid concentrations

A mixture of 80% acetone and anhydrous ethanol (1:1) was used to completely extract the photosynthetic pigments from fresh leaf samples (0.1 g). Using a spectrophotometer (Shimadzu UV-1900 Kyoto, Japan), the absorbances at 440, 645, and 663 nm were read for the determination of carotenoids, chlorophyll a, and chlorophyll, respectively [35]. Their concentrations were determined using the following equations:

2 $$\mathrm{Chlorophyll}\;\mathrm a\;(\mathrm{Chl}\;\mathrm a)\;=\;9.784\mathrm A663\;-\;0.990\mathrm A645$$

3 $$\mathrm{Chlorophyll}\;\mathrm b\;(\mathrm{Chl}\;\mathrm b)\;=\;21.426\mathrm A645\;-\;4.650\mathrm A663$$

4 $$\mathrm{Carotenoids}\;(\mathrm{Car})\;=\;4.695\mathrm A440\;-\;0.268\mathrm{Chl}\;\mathrm t$$

### Estimation of mineral elements

Dried leaf samples (0.05 g) were treated with 4 ml of deionized water for 40 min at 100 °C and centrifuged for 15 min at 3000 *× g*. In the next step, the supernatant was collected in tubes, and used an inductively coupled plasma atomic emission spectrometer to determine the concentrations of K^+^, Mg^2+^, Na^+^, Fe^2+^, B^3+^, Zn^2+^, Mn^2+^, and Ca^2+^. Additionally, ion chromatography was conducted (DX-300 ion chromatography system, CDM-II electrical conductivity detector, AS4A-SC chromatographic column, mobile phase: Na_2_CO_3_/NaHCO_3_ = 1.7/1.8 mM, DIONEX, Sunnyvale, U.S.A.) to determine the Cl^−^ concentration.

### Determination of oxidative stress indicators and antioxidant mechanisms

The concentration of hydrogen peroxide (H_2_O_2_) was estimated using a standard protocol [36]. Fresh samples were homogenized in 5.0 ml of 0.1% trichloroacetic acid (TCA) and centrifuged for 15 min at 12,000 *× g*. The absorbance was read at 390 nm using a spectrophotometer to determine the H_2_O_2_ concentration. Moreover, malondialdehyde (MDA) was measured using the thiobarbituric acid (TBA) test [37]. Fresh leaf samples (0.5 g) were homogenized in 5% trichloroacetic acid (TCA) solution for 10 min at 4 °C, followed by centrifugation at 5,000 *× g* for 10 min and the addition of 20% TCA to the mixture. Afterwards, the mixture was heated at 100 °C (15 min) and centrifuged at 5,000 *× g* for 15 min. Afterward, the absorbances were read at 450, 532, and 600 nm using a spectrophotometer. Moreover, fresh leaf samples were homogenized in 5% sulfosalicylic acid to determine the concentrations of ascorbate (AsA), dehydroascorbate (DHA), glutathione (GSH), and oxidized glutathione (GSSG) [38].

### Estimation of antioxidant enzyme activities

Fresh leaf samples were homogenized using phosphate buffer (50 mM, pH 7.8) and EDTA-Na_2_O (0.1 mM) and centrifuged at 10,000 *× g* at 4 °C for 5 min. The supernatants were subsequently collected and used to assess enzymatic activity. The activity of superoxide dismutase (SOD; E.C.1.15.1.1) was determined by measuring its absorbance at 560 nm to determine whether enzyme extracts can prevent the photochemical degradation of nitroblue tetrazolium (NBT) [39]. The peroxidase (POD) activity was measured by standard protocol (Wang et al., 2018), but with a few minor modifications. Two readings at 460 nm were taken at intervals of one minute each. The enzyme activity was calculated in units of U/g per minute [40]. Catalase (CAT) activity was determined as described in a previous report by measuring the absorbance at 240 nm. CAT activity was defined as the amount of CAT required to decompose H_2_O_2_ (1.0 µM) [41]. The activity of ascorbate-glutathione cycle enzymes, including ascorbate peroxidase (APX), glutathione reductase (GR), monodehydroascorbate reductase (MDHR), and dehydroascorbate reductase (DHAR), was estimated by measuring the changes in absorbances at 290 nm, 340 nm, 340 nm, 265 nm, and 340 nm, respectively [36].

### Biochemical determination

The proline content was estimated using the method of Bates et al. [42]. Samples of fresh leaves (0.5 g) were homogenized in 5 ml (3%) of aqueous sulfosalicylic acid. In the following step, the homogenate was centrifuged for 12 min at 11,500 *× g*. The supernatant was then thoroughly mixed with acid ninhydrin and glacial acetic acid. The reaction mixture was subsequently boiled for one hour at 100 °C and cooled. With the addition of 2 ml of toluene, the red color was removed from the chromophore, and the absorbance at 520 nm was measured using a spectrophotometer (Beckman 640 D, USA). The concentration of glycine betaine was determined using a standard protocol [43]. The concentration of soluble sugar was determined according to a previous method. Glucose served as a standard for the calculation [44]. Additionally, leaf samples were homogenized in phosphate buffer (pH 7.0) with Coomassie brilliant blue G-250 at 595 nm to detect soluble proteins via spectrophotometry. Bovine serum albumin (BSA) solutions were used to construct the standard curve [45].

### Statistical analysis

Descriptive statistics and one-way analysis of variance (ANOVA) were performed using SPSS version 16.0 (Chicago, IL, USA). Significance was determined at a threshold of *p* < 0.05, and Duncan’s test was employed for mean comparisons. GraphPad Prism 8 was used to create the graphics. To analyze growth and physiological parameters, a Pearson correlation analysis was conducted using OriginPro 2019 software (Origin Lab Corporation Northampton, Northampton, MA, USA). OriginPro 2019 software was used to perform a principal component analysis (PCA) of the variables. In PCA, relationships between variables can be observed. As we analyzed each variable separately in the ANOVAs, we were able to observe qualitatively the similarities and differences between treatments when taking all variables into account together in the PCA.

## Results

### Changes in growth parameters

The growth performance of soybean plants was adversely impacted by the imposition of saline stress. compared to the control (Table 1). The extent of inhibition varied according to the salt concentration, with higher levels of inhibition noted at 80 mM, followed by 40 mM. For example, both saline stress levels (40- and 80 mM), significantly decreased the shoot height, root length, and the fresh and dry weight of root, stem, and leaves compared to the control (0 mM). Conversely, the root/shoot (R/S) ratio remained nonsignificant under 40 mM SS but significantly increased under 80 mM stress (Table 1).

**Table 1:** Changes in growth characteristics of soybean seedlings under saline stress and GABA application

Parameters	Control (0-mM saline stress)		40-mM saline stress		80-mM saline stress	
	-GABA	+GABA	-GABA	+GABA	-GABA	+GABA
**SL (cm)**	22.59 ± 1.63 b	29.59 ± 2.21 a	16.23 ± 1.00 d	19.09 ± 0.80 c	12.03 ± 1.21 e	13.51 ± 1.00 e
**RL (cm)**	14.93 ± 0.59 ab	16.09 ± 0.86 a	11.95 ± 0.44 c	14.53 ± 0.72 b	9.48 ± 0.57 d	11.45 ± 0.97 c
**SFW (g)**	3.56 ± 0.15 b	4.39 ± 0.17 a	2.57 ± 0.12 c	3.22 ± 0.31 b	1.30 ± 0.23 e	1.70 ± 0.13 d
**RFW (g)**	1.64 ± 0.08 a	1.80 ± 0.06 a	1.31 ± 0.09 b	1.73 ± 0.16 a	0.73 ± 0.05 c	0.85 ± 0.09 c
**LFW (g)**	5.74 ± 0.38 b	7.34 ± 0.30 a	3.67 ± 0.39 d	4.79 ± 0.59 c	2.24 ± 0.26 f	2.93 ± 0.49 e
**SDW (g)**	1.34 ± 0.03 b	1.68 ± 0.09 a	1.07 ± 0.14 c	1.26 ± 0.10 b	0.57 ± 0.07 d	0.73 ± 0.12 d
**RDW (g)**	0.58 ± 0.06 b	0.66 ± 0.03 a	0.42 ± 0.03 cd	0.47 ± 0.02 c	0.39 ± 0.04 e	0.43 ± 0.06 cd
**LDW (g)**	3.25 ± 0.16 a	3.34 ± 0.12 a	2.44 ± 0.08 c	2.96 ± 0.20 b	1.32 ± 0.14 e	1.85 ± 0.14 d
**RSR**	0.13 ± 0.02 c	0.13 ± 0.01 c	0.12 ± 0.02 c	0.12 ± 0.01 c	0.21 ± 0.02 a	0.18 ± 0.03 b

The different letters indicate significant differences between the values at *P* < 0.05 (Duncan method). *SL *Shoot length, *RL *Root length, *SFW *Stem fresh weight, *RFW* Root fresh weight), *LFW* Leaves fresh weight), *SDW* Stem dry weight), *RDW* Root dry weight), and *LDW* Leaves dry weight. 0 mM, 40 mM, and 80 mM represent different concentrations of saline stress

Exogenous GABA application led to improvements in various growth parameters under different saline stress levels, albeit to varying extents (Table 1). For instance, it significantly enhanced the shoot height, root fresh weight, and shoot dry weight under 40 mM stress level, while exhibiting little improvements under 80 mM stress, compared to their untreated GABA peers (Table 1). On the other hand, root length, leaf and stem fresh weight, as well as leaf and root dry weight significantly improved under both 40 mM and 80 mM stress levels following GABA supplementation. Under control conditions, GABA application also resulted in improvements in shoot height, stem fresh weight, and dry weights of leaves, stems, and roots (Table 1).

### Changes in photosynthetic pigments

**Fig. 1:**
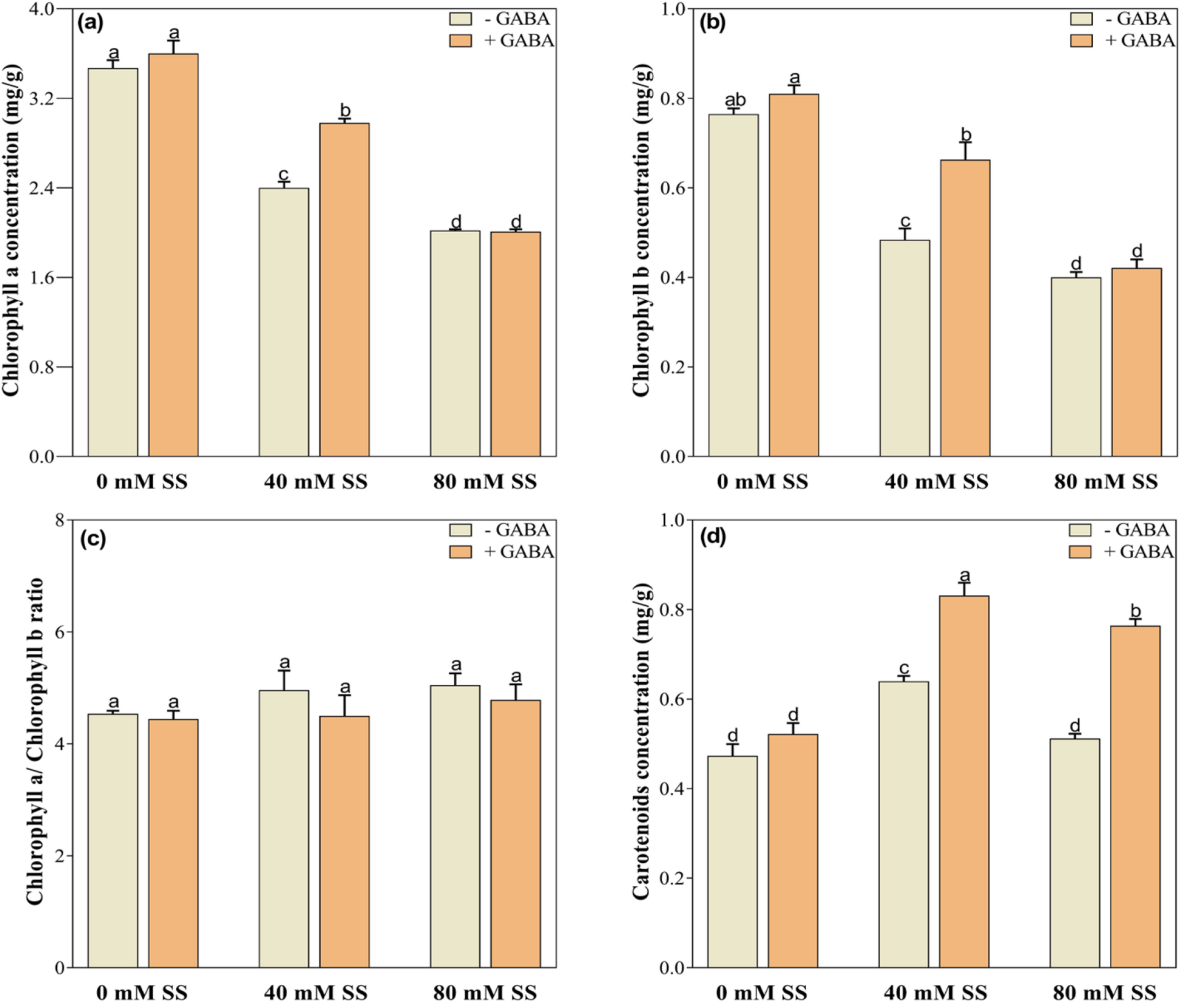
Changes in the concentrations of (**a**) chlorophyll a, (**b**) chlorophyll b, (**c**) chlorophyll a/chlorophyll b, and (**d**) carotenoids under saline stress and GABA application. The graphs indicate the mean and standard deviation (mean ± SD) of the data. The different letters above the bars indicate significant differences between the values at *P* < 0.05 (Duncan method). 0 mM, 40 mM, and 80 mM represent different concentrations of saline stress

Salinity stress influenced the concentration of photosynthetic pigments, resulting in significant inhibition of chlorophyll a and b concentrations under both 40- and 80-mM stress levels, compared to control (Fig. 1a, b). The chlorophyll a/chlorophyll b (Chl a/b) ratio demonstrated little changes under both stress levels, regardless of GABA application (Fig. 1c). Carotenoid (Car) concentrations increased under 40 mM saline stress while exhibited little increase under 80 mM stress (Fig. 1d). In comparison to their untreated peers, exogenous GABA significantly increased Chl and Chl b under 40 mM stress while Car increased under both 40- and 80-mM treated soybean seedlings (Fig. 1a, b, d).

### Changes in mineral nutrients

**Fig. 2:**
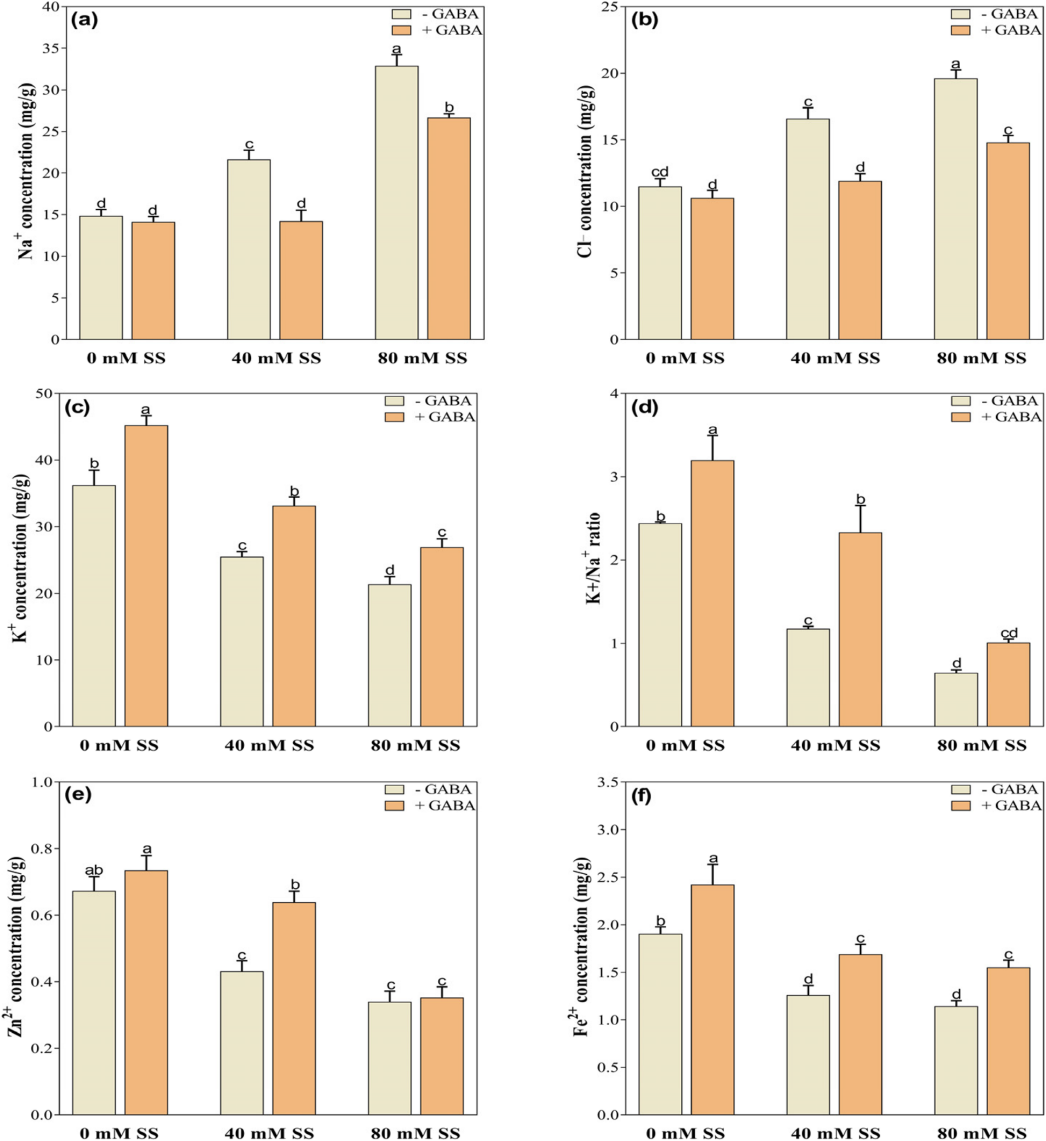
Changes in the concentrations of (**a**) Na^+^(**b**) Cl^−^(**c**) K^+^(**d**) K^+^/Na^+^ratio, (**e**) Zn^2+,^and (**f**) Fe^2+^under saline stress and GABA application. The graphs indicate the mean and standard deviation (mean ± SD) of the data. The different letters above the bars indicate significant differences between the values at *P* < 0.05 (Duncan method). 0 mM, 40 mM, and 80 mM represent different concentrations of saline stress

**Fig. 3:**
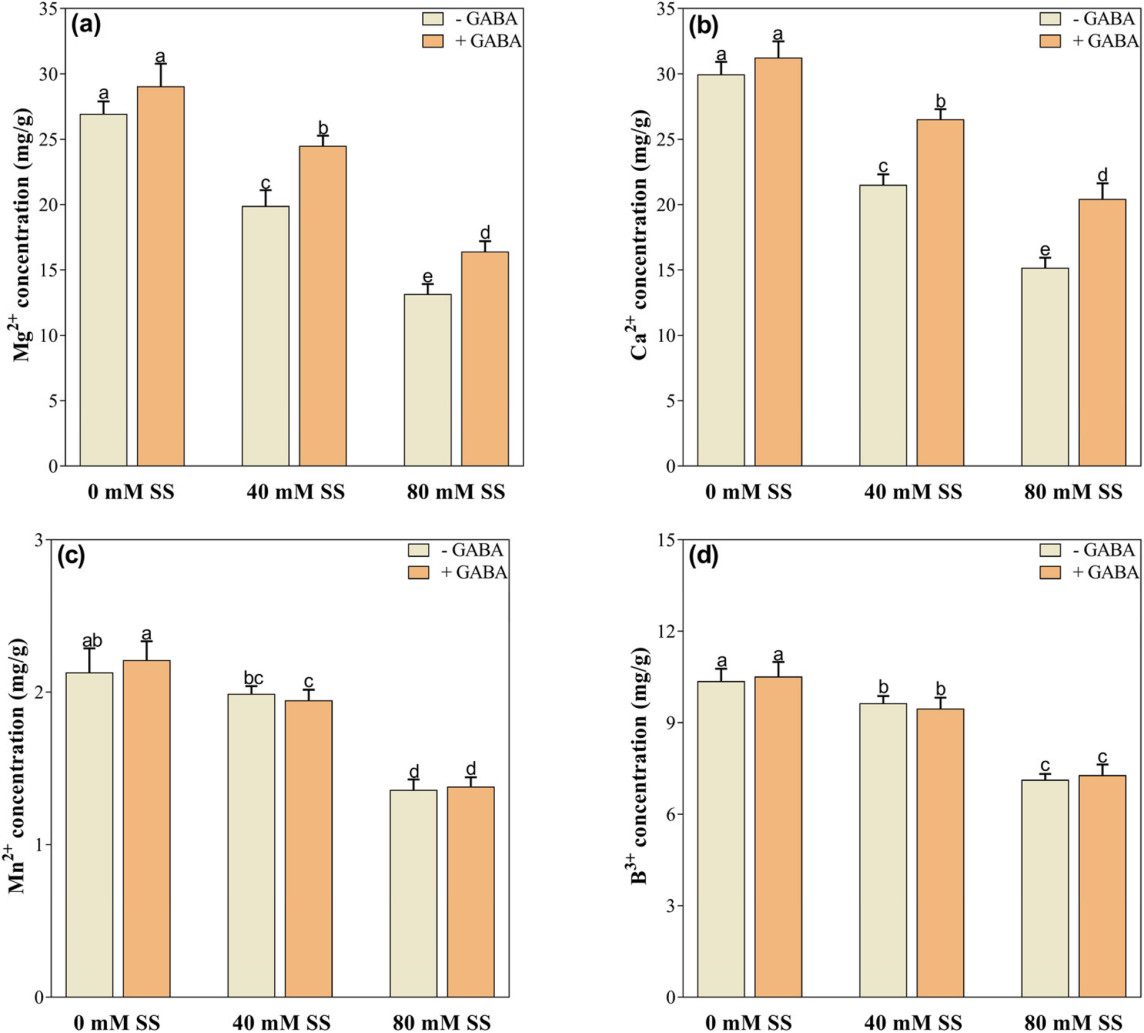
Changes in the concentrations of (**A**) Mg^2+^(**B**) Ca^2+^(**C**) Mn^2+^and (**D**) B^3+^under saline stress and GABA application. The graphs indicate the mean and standard deviation (mean ± SD) of the data. The different letters above the bars indicate significant differences between the values at *P* < 0.05 (Duncan method). 0 mM, 40 mM, and 80 mM represent different concentrations of saline stress

Salinity stress also affected mineral nutrient accumulation, notably increasing Na^+^ and Cl^−^ concentrations while decreasing K^+^, K^+^/Na^+^, Zn^2+^, Fe^2+^, Mg^2+^, and Ca^2+^ and B^3+^ concentrations under both stress levels, compared to control. However, Mn^2+^ significantly reduced under 80 mM but exhibited little changes under 40 mM stress (Figs. 2a-f and 3a-d).

However, GABA treatment alleviated the adverse effects of saline stress on nutrient accumulation to varying degrees. It significantly reduced Na^+^ and Cl^-^ under both stress levels, compared to their untreated peers. Conversely, GABA application improved K^+^, Fe^2+^, Mg^2+^, and Ca^2+^, under both stress levels whereas Zn^2+^, Mn^2+^, and B^3+^ as well as K^+^/Na^+^ ratio only under 40 mM saline stress, compared to their untreated GABA counterparts (Figs. 2a-f and 3a-d).

### Changes in osmolyte production

**Fig. 4:**
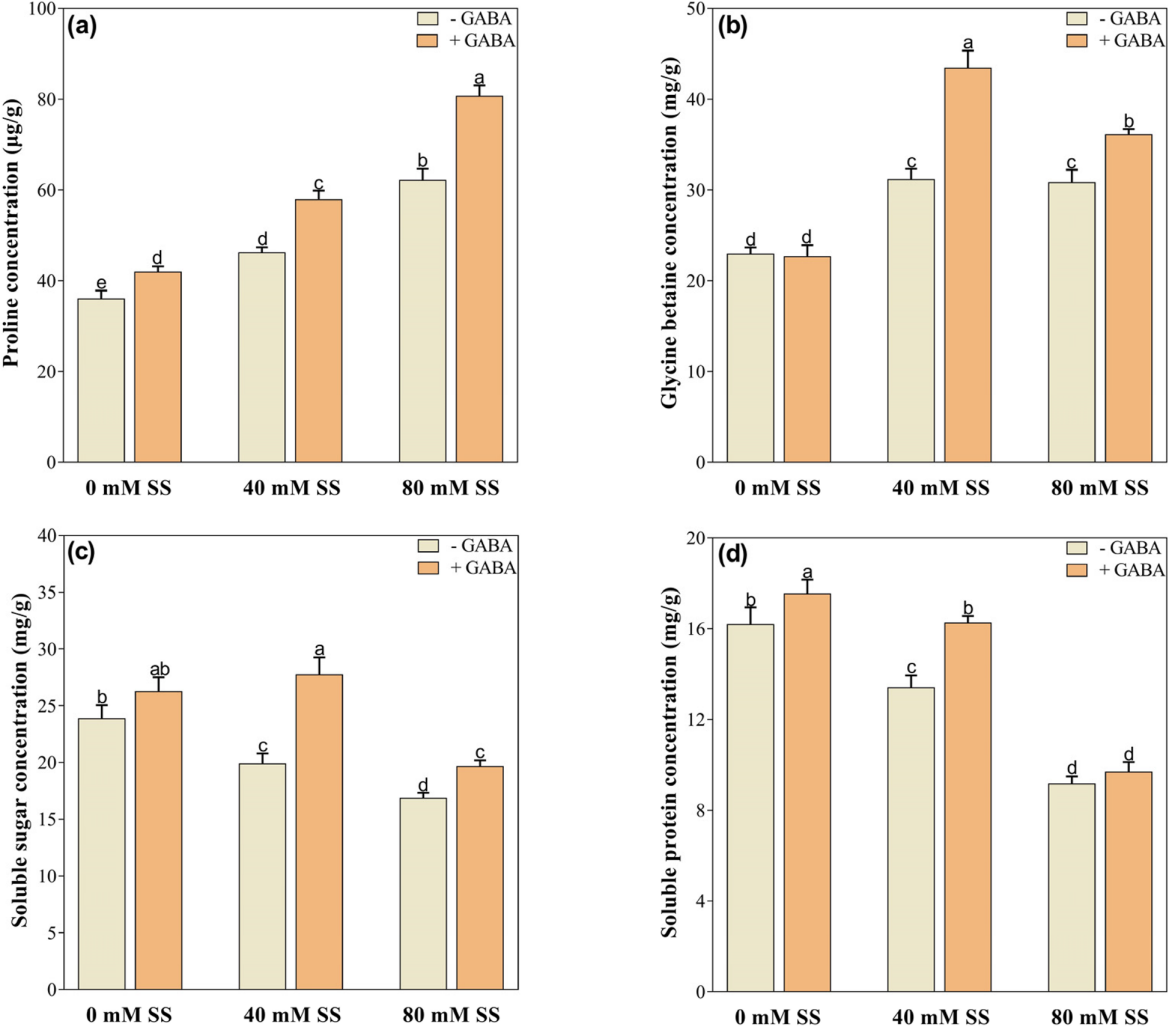
Changes in the concentrations of (**a**) proline, (**b**) glycine betaine, (**c**) soluble sugar, and (**d**) soluble protein under saline stress and GABA application. The graphs indicate the mean and standard deviation (mean ± SD) of the data. The different letters above the bars indicate significant differences between the values at *P* < 0.05 (Duncan method). 0 mM, 40 mM, and 80 mM represent different concentrations of saline stress

The concentrations of osmolytes such as proline and glycine betaine increased under both 40- and 80-mM saline stress, compared to the control (Fig. 4a, b). However, soluble sugar and soluble protein contents decreased significantly, under either stress, regardless of GABA application (Fig. 4c, d). GABA application further enhanced the concentrations of osmolytes and soluble proteins. However, GABA treatments increased the concentrations of proline, glycine betaine, and soluble sugar under both stress levels and soluble proteins under 40 mM stress, compared to their untreated GABA peers (Fig. 4a, b).

In contrast, these strains exhibited lower soluble sugar (16 and 29%) and soluble protein (17 and 43%) contents (Fig. 4c, d). Similarly, compared with the untreated GABA counterparts, the GABA treatment improved the proline, glycine betaine, and soluble sugar contents by 20 and 29%, 39 and 17%, and 42 and 17%, respectively. Furthermore, soluble proteins increased by 21% under 40 mM stress (Figure a-d).

### Oxidative stress indicators

**Fig. 5:**
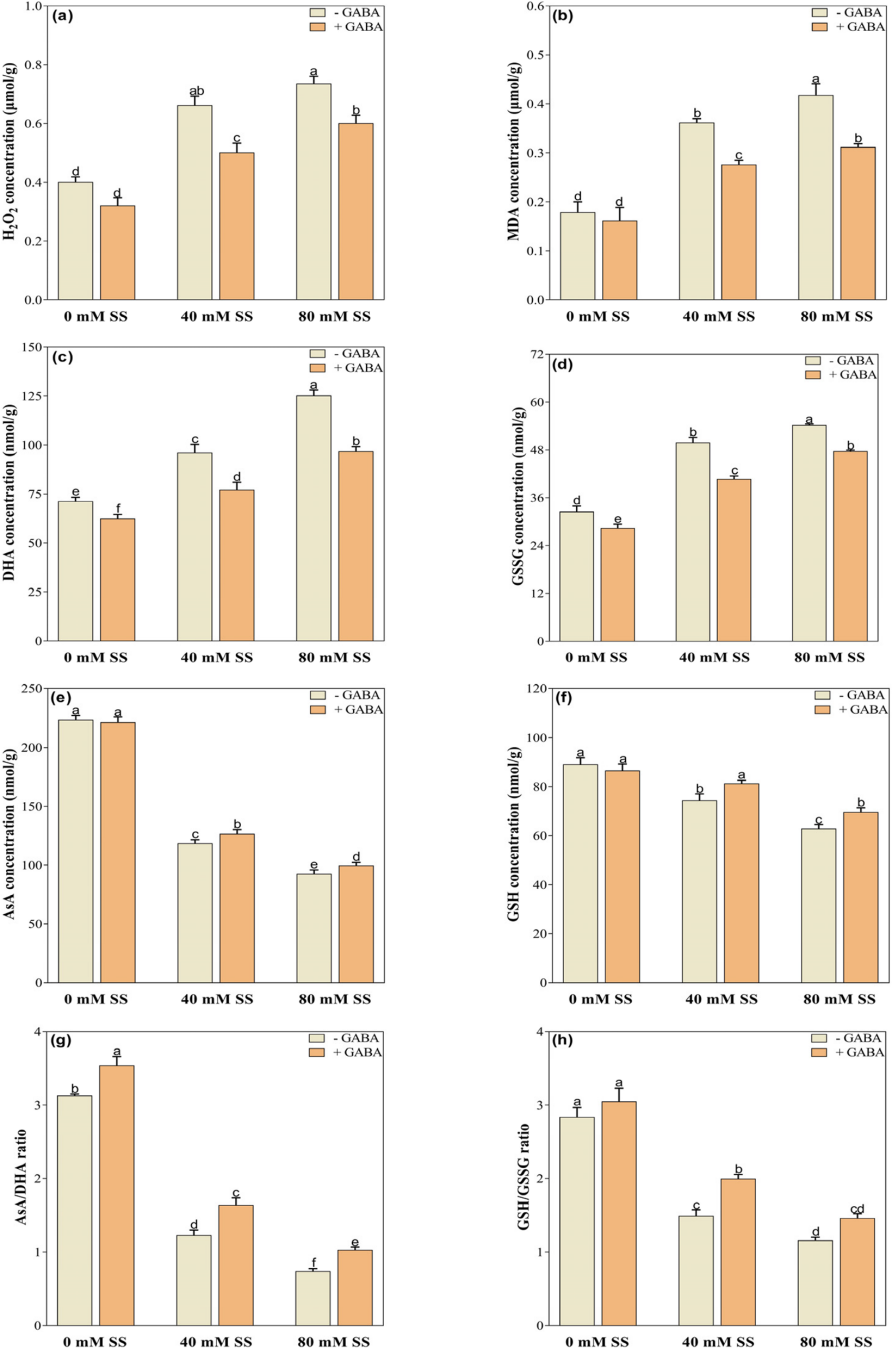
Changes in the concentrations of **a** H_2_O_2_, **b** MDA, **c** DHA, **d** GSSG, **e** AsA, **f** GSH, **g** AsA/DHA and **h** GSH/GSSG under saline stress and GABA application. The graphs indicate the mean and standard deviation (mean ± SD) of the data. The different letters above the bars indicate significant differences between the values at *P* < 0.05 (Duncan method). 0 mM, 40 mM, and 80 mM represent different concentrations of saline stress

Oxidative stress indicators, including H_2_O_2_, MDA, DHA, and GSSG, increased in soybean seedlings exposed to both 40- and 80-mM saline stress levels, compared to control (Fig. 5a-d). Conversely, AsA and GSH concentrations decreased significantly, resulting in lower AsA/DHA and GSH/GSSG ratios (Fig. 5e-h). However, GABA treatment mitigated oxidative stress by reducing the concentrations of oxidative stress biomarkers. For instance, GABA-treated seedlings exhibited lower H_2_O_2_, MDA, DHA, and GSSG under both saline stress levels, compared to their untreated GABA peers (Fig. 5a-d). However, it increased AsA under 40 mM and GSH under both 40- and 80-mM stress levels, resulting in improved AsA/DHA and GSH/GSSG ratios (Figure e-h). Under control conditions, GABA application had no significant effect on H_2_O_2_, MDA, AsA, GSH, and GSH/GSSG ratios (Fig. 5a, b. e, f, h). However, it significantly decreased DHA and GSSG but improved the GSSG ratio (Fig. 5, c, d, g).

### Changes in the activity of antioxidant enzymes

**Fig. 6:**
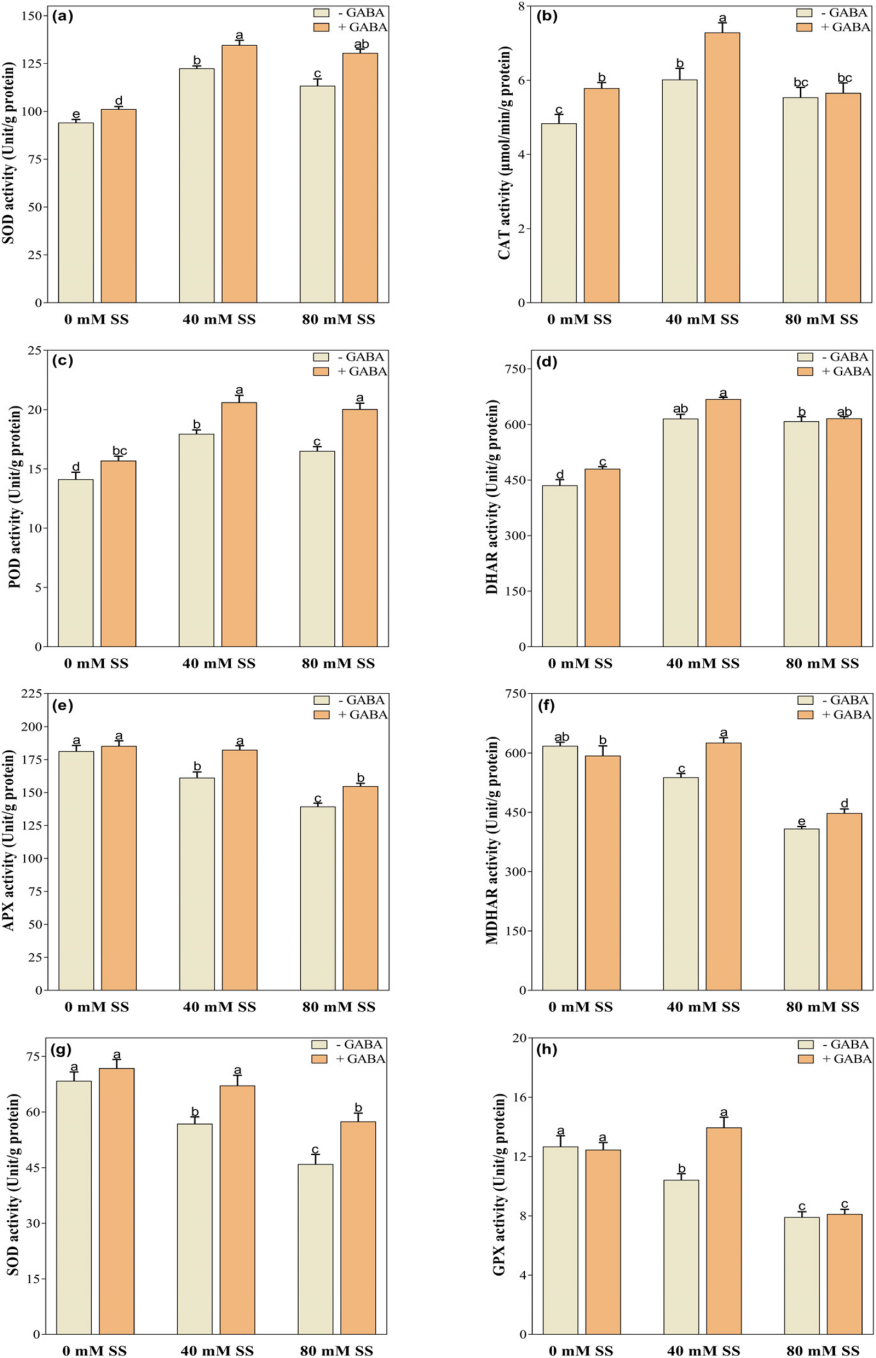
Changes in the enzymatic activity of **a** SOD, **b** CAT, **c** POD, **d** DHAR, **e** APX, **f** MDHAR, **g** GR, and **h** GPX under saline stress and GABA application. The graphs indicate the mean and standard deviation (mean ± SD) of the data. The different letters above the bars indicate significant differences between the values at *P* < 0.05 (Duncan method). 0 mM, 40 mM, and 80 mM represent different concentrations of saline stress

The enzymatic activity of antioxidant enzymes SOD, CAT, POD, and DHAR increased under both stress levels, compared to the control, regardless of GABA application (Fig. 6a-d). Conversely, under both 40- and 80-mM stress, the activities of APX, MDHAR, GR, and GPX decreased, decreased significantly (Fig. 6g-h). However, GABA treatment enhanced the enzymatic activities of antioxidant enzymes, indicating its role in mitigating oxidative stress under saline stress. For example, SOD, POD, APX, MDHAR, and GR are under both stress levels while CAT and GPX are under 40 mM stress, compared to their untreated peers (Fig. 6a-h). Moreover, it also enhanced SOD, CAT, POD, and DHAR under control conditions.

### Pearson correlation and principal component analysis

**Fig. 7:**
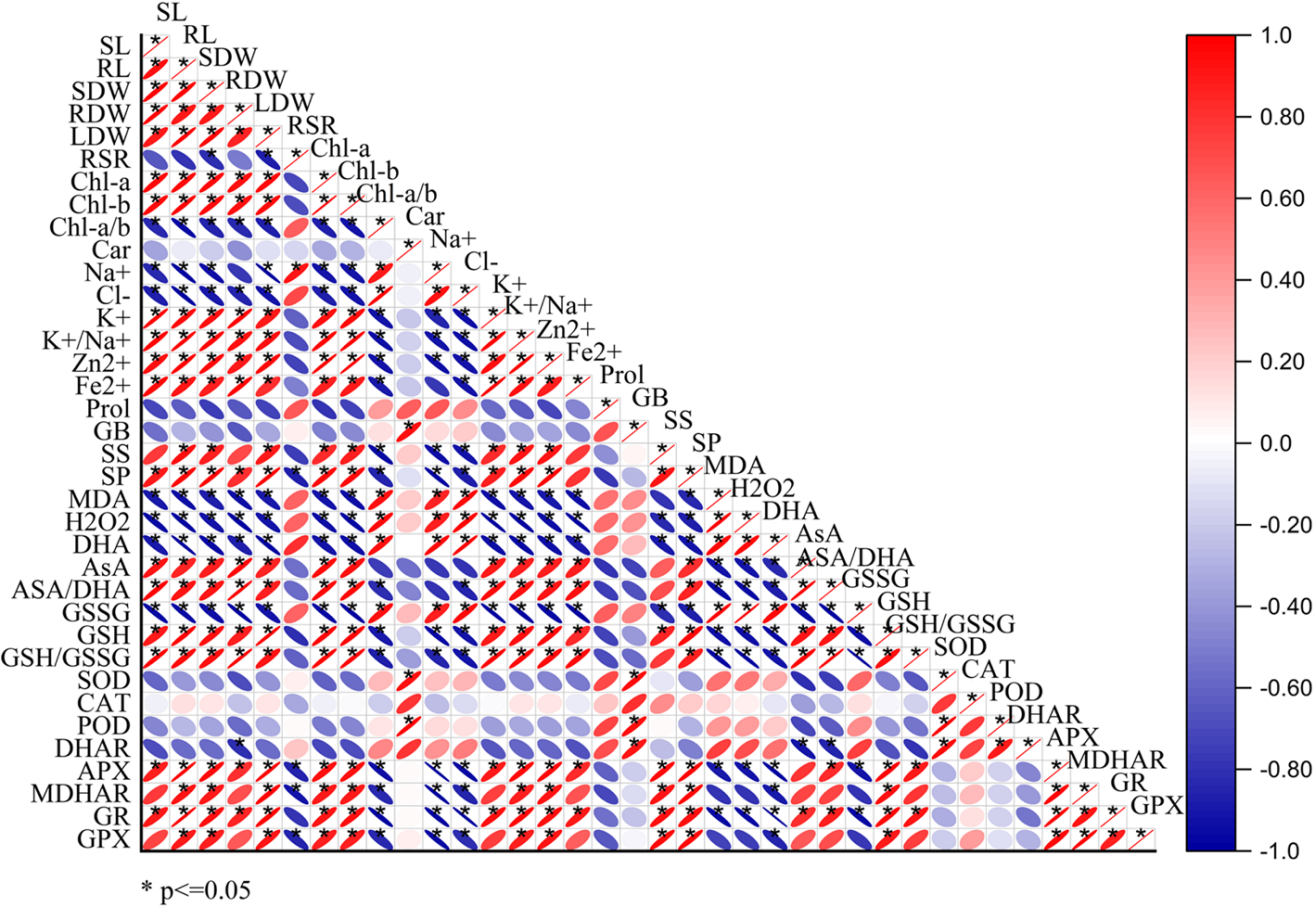
Pearson correlation of the studied morphological and physio-biochemical parameters. Shoot length (SL), root length (RL), shoot dry weight (SDW), shoot dry weight (RDW), leaf dry weight (LDW), root/shoot ratio (R/S), chlorophyll a (Chl a), chlorophyll b (Chl b), chlorophyll a/chlorophyll a (Chl a/b), carotenoids (Car), malondialdehyde (MDA), hydrogen peroxide (H_2_O_2_), ascorbate (AsA), dehydroascorbate (DHA), glutathione (GSH), oxidized glutathione (GSSG), ascorbate peroxidase (APX), monodehydroascorbate reductase (MDHAR), dehydroascorbate reductase (DHAR), glutathione reductase (GR), glutathione peroxidase (GPX), superoxide dismutase 9SOD0, peroxidase (POD), catalase (CAT), proline (Pro), soluble sugar (SS), soluble protein (SP), and glycine betaine (GB) were measured

According to the Pearson correlation analysis, the accumulation of Na^+^ and Cl^−^ was negatively correlated with the following study growth characteristics: minerals (K^+^, K^+^/Na^+^, Ca^2+^, Mg^2+^, Fe^2+^, Mn^2+^, B^3+^, and Zn^2+^); ascorbate-glutathione metabolites (AsA, GSH, AsA/DHA and GSH/GSSG); enzymes (APX, MDHAR, GR and GPX); and osmolytes (soluble proteins and soluble sugars) (Fig. 7).

In contrast, they showed no significant correlation with CAT or POD activity and an insignificant negative correlation with Car. Furthermore, there were strong positive correlations between the R/S ratio, Chl a/b, oxidative status (H_2_O_2_, MDA, DHA, and GSSG), antioxidant enzymes (SOD and GR), and osmolytes (Pro and GB) (Fig. 7).

**Fig. 8:**
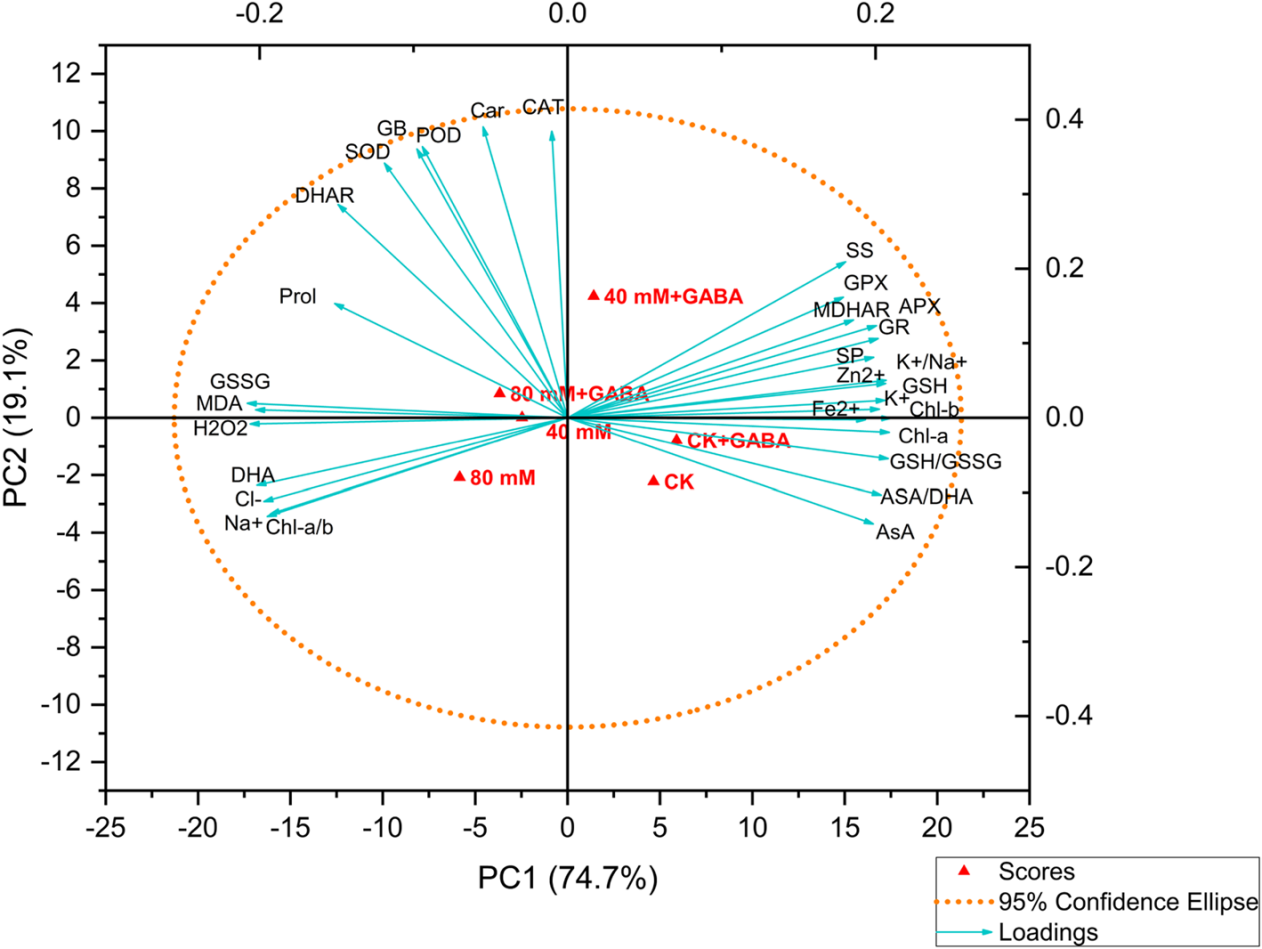
Principal component analysis of the studied morphological and physio-biochemical parameters. Shoot dry weight (SDW), root dry weight (RDW), leaf dry weight (LDW), the root/shoot weight ratio (R/S), the chlorophyll a (Chl a), the chlorophyll b (Chl b), the chlorophyll a/chlorophyll a (Chl a/b), carotenoids (Car), malondialdehyde (MDA), hydrogen peroxide (H_2_O_2_), ascorbate (AsA), dehydroascorbate (DHA), glutathione (GSH), oxidized glutathione (GSSG), ascorbate peroxidase (APX), monodehydroascorbate reductase (MDHAR), dehydroascorbate reductase (DHAR), glutathione reductase (GR), glutathione peroxidase (GPX), superoxide dismutase 9SOD0, peroxidase (POD), catalase (CAT), proline (Pro), soluble sugar (SS), soluble protein (SP), and glycine betaine (GB) were measured

A Principal Component Analysis (PCA) was conducted to examine the variability of the collected data and the relationships between the different treatments and attributes. The analysis revealed that PC1 and PC2 together explain 93.8% of the total variability in the data resulting from diverse treatments. PC1 accounted for 65.7% of the variation, while PC2 contributed to 24.5% of the total variation. The biplot was divided into clusters. The oxidative stress indicators, such as H_2_O_2_, MDA, GSSG, and DHA, as well as Na^+^, Cl^−^, and Chl-a/b ratio, were clustered together and were proximate to the 40- and 80-mM stress treatment. On the other hand, mineral nutrients, chlorophyll pigments, AsA, GSH, and their metabolizing enzymes, were clustered together. Moreover, osmolytes such as proline and GB, and H_2_O_2_ scavenging mechanisms such as SOD, POD CAT, and DHAR, were placed amid oxidative stress biomarkers and minerals, pigments, and the AsA-GSH cycle. The PCA plot revealed a positive correlation among the parameters related to the H_2_O_2_ elimination mechanism, osmolytes, and the activity of GPX, GR, MDHAR, and APX enzymes, as well as the AsA-GSH metabolites and the content of chlorophyll pigments and mineral ions (Fig. 8). Conversely, there was an unfavorable correlation observed among plant mineral nutrition, the AsA-GSH cycle, pigments, soluble sugars, proteins, and oxidative stress biomarkers. Osmolytes and ROS-eliminating enzymes were positioned amidst these variables, indicating their role in alleviating salinity stress. Notably, treatments involving 80 mM and 40 mM salinity were associated with oxidative stress biomarkers. However, GABA application under salinity conditions showed a strong association with antioxidants, suggesting the efficacy of GABA supplementation in mitigating salinity stress (Fig. 8).

## Discussion

### GABA-enhanced growth and pigments under salinity

In our study soybean plants subjected to saline stress exhibited a decrease in shoot height, root length, and leaf-stem and root fresh and dry weight, compared to the control (Table 1). Growth and biomass are negatively correlated with high salt ions (Na^+^ and Cl^−^) and oxidative stress biomarkers (H_2_O_2_ and MDA) (Fig. 7). Excessive salt ions such as Na^+^ and Cl^−^ interfere with the uptake of K^+^, Mg^2+^, and Ca^2+^, causing severe water loss and cellular necrosis [46, 47]. Furthermore, excessive salt ions alter metabolism and enzymatic functions, resulting in the production of higher ROS, which causes oxidative stress and impairs plant performance [48]. Hence, our results revealed that salinity inhibits plant growth by modulating several mechanisms. These mechanisms include excessive accumulation of ROS, ionic toxicity [9, 49], osmotic stress [50], impaired photosynthesis [51], and lower mineral nutrient uptake and cell division [52, 53], resulting in reduced growth and biomass production.

Furthermore, salinity significantly reduced the concentrations of chlorophyll a, chlorophyll b, and carotenoid, compared to control (Fig. 1a-d). Photosynthesis requires an optimal concentration of chlorophyll. In saline conditions, plant growth is typically reduced by a decline in photosynthesis. As a result of a series of stepwise reactions, chlorophyll biosynthesis may be disrupted, resulting in reduced chlorophyll concentration [30, 54, 55], net photosynthesis, and biomass production. The salinity-induced reductions in chlorophyll and carotenoids may be attributed to oxidative damage-induced membrane breakdown, and chlorophyllase which negatively impacts pigment synthesis [56, 57].

Endogenous GABA can improve a plant’s ability to cope with stress [58]. Exogenous GABA could increase the levels of endogenous GABA within plant tissues, which could result in an increase in tolerance to salinity stress. In our study, GABA improved pigment synthesis and growth in soybean seedlings under salinity compared to GABA-untreated seedlings. These findings are in agreement with those reported by Jin et al. [29] and Ullah et al. [24], who reported that exogenous GABA positively modulated the physiological mechanisms and growth of watermelon and mungbean plants under saline stress, respectively. It has been reported that GABA positively regulates chlorophyll synthesis, stomata regulation, and intercellular CO_2_, and reduces oxidative stress damage [59], which agrees with our results. Hence, we suggest that GABA has the potential to alleviate the detrimental impacts of salinity on the growth parameters and chlorophyll pigments in soybeans resulting in improved tolerance and growth of soybeans [30, 60, 61].

### GABA reduced ROS and improved antioxidant potential under salinity

As a result of abiotic stress, several biochemical markers (i.e., ROS, O_2_^−^, and H_2_O_2_) are released in plants and serve as signaling molecules for plant defense mechanisms [62]. Nevertheless, the excessive production of these markers has the potential to adversely affect some biochemical and physiological processes in plants [63]. In our study, increased SS levels significantly elevated the concentrations of H_2_O_2_ and MDA, suggesting the phenomenon of oxidative stress damage in soybean seedlings [30, 47, 61], compared to the control. Excessive ROS levels cause the degradation of lipids, proteins, and DNA, resulting in cellular leakage and death [4]. Furthermore, salinity stress can degrade the photosynthetic apparatus and elevate levels of abscisic acid, leading to stomatal closure, decreased net photosynthetic rates, and lower biomass production [4, 64]. Several antioxidant enzymes such as SOD, APX, POD, and CAT, are known to eliminate ROS and protect cells from oxidative damage [56, 65, 66]. The enzyme SOD is responsible for converting O_2_^−^ to H_2_O_2_, whereas POD, CAT, and APX convert H_2_O_2_ to H_2_O and O_2_ [67]. In our study, salinity stress significantly increased the activity of SOD, CAT, and POD but decreased GPX, compared to the control (Fig. 6a-d), suggesting that soybean seedlings were able to reduce the salinity-induced oxidative damage through enhanced antioxidant enzymes, for removing O_2_^−^ to H_2_O_2_. However, this response was accompanied by a substantial reduction in biomass production.

As non-enzymatic molecules, both AsA and GSH play a crucial role in the antioxidant mechanism as components of the AsA-GSH cycle. The enzyme APX oxidizes AsA to MDHA, which is then converted to DHA through the removal of H_2_O_2_. In addition, MDHA and DHA can be converted back to AsA through the action of MDHAR and DHAR respectively. Moreover, the GR enzyme regenerates GSH from GSSG [12]. However, the concentration of AsA and GSH may vary under stress, but their redox buffering functions can initiate stress acclimation processes [68]. AsA and GSH pools are reduced under salinity stress [69, 70]. This could be attributed to the impaired enzymatic activities related to the AsA-GSH cycle. In our study, exposure to salinity stress resulted in higher DHAR activity but decreased APX, MDHAR, and GR, indicating the susceptibility and limited efficacy of the AsA-GSH cycle in neutralizing H_2_O_2_ in soybean seedlings.

According to both this study and previous research [29, 56], exogenous GABA stimulates antioxidant mechanism in plants to efficiently scavenge H_2_O_2_. For instance, GABA additions have been shown to regulate SOD, POD, and CAT and the enzymes of the AsA-GSH cycle in some crops, including tomatoes [71] and mungbean [24], and chufa [30]. Moreover, GABA application also increased GPX activity in salinity-stress soybeans, compared to their untreated GABA peers. GPX has a multitude of physiological functions, such as oxidizing toxic compounds, synthesizing cell walls, and regulating plant growth under stress conditions [72]. Likewise, another study on tomato seedlings has also demonstrated significantly higher activity of SOD, POD, and CAT, which has been shown to result in lower ROS production and lower oxidative stress under salinity [73]. In our study, we observed a similar scenario, where the application of exogenous GABA enhanced antioxidant activity and maintained ROS metabolism balance. Moreover, our results indicate that exogenous GABA also mitigates ion toxicity and minimizes oxidative damage, both linked to increased antioxidant enzyme activity, thereby restoring plant growth.

### GABA application positively regulated ions in soybeans under salinity

In plants subjected to salinity stress, excessive Na^+,^ and Cl^−^ accumulation leads to a decrease in the uptake of essential nutrients [51, 74] and the modulation of essential physiological mechanisms. For example, increasing Na^+^ decreases K^+^, which may degrade chlorophyll, disrupt thylakoids [75], and ultimately impair photosynthetic activity and other enzymatic functions. Hence, the ability of plants to tolerate salinity is reflected in their ability to reduce the uptake of Na^+^ ions in cells [76]. Our results indicate that increased salinity stress resulted in significantly greater concentrations of Na^+^ and Cl^−^ but decreased concentrations of K^+^, Mg^2+^, Ca^2+^, Zn^2+^, and Fe^2+^ compared to control. Furthermore, salinity-induced decreases in K^+^ ions are associated with low K^+^/Na^+^ ratios (Fig. 3c, d). High salinity in the rhizosphere results in high pH, which reduces the availability of mineral ions and hence negatively impacts physiological mechanisms and growth [51]. The repression of K^+^ absorption by stress may also contribute to low K^+^ concentration. Therefore, K^+^ and Na^+^ might compete for binding sites for cellular functions due to the low K^+^/Na^+^ ratio [77]. We suggest that the decrease in beneficial mineral nutrients in soybeans could be explained by cellular membrane damage caused by ionic toxicity, osmotic imbalance, and pH-induced damage because of the high accumulation of Na^+^ and Cl^−^ ions [56, 78, 79]. Therefore, we suggest that increased salt ions and decreased beneficial nutrients resulting from salinity may result in nutrient imbalances in soybean seedlings, resulting in reduced dry matter accumulation and reduced growth, as demonstrated by previous research findings on soybean [51], canola [80], barley [81], and maize [56].

Compared to untreated plants, GABA application significantly suppressed Na^+^ and Cl^-^ ions while enhancing the concentrations of beneficial ions such as K^+^, Fe^2+^, Mg^2+^, Ca^2+,^ and the K^+^/Na^+^ ratio under both stress conditions. Additionally, Zn^2+^ improved only under 40 mM stress. The effect of GABA on plant Na^+^ and Cl concentrations has been extensively studied; however, it is unclear whether GABA can directly reduce the accumulation of toxic salt ions. GABA may reduce toxic salts under saline conditions due to its osmotic regulatory mechanism, which mitigates stress damage, resulting in the normalization of essential ion uptake [82]. It has been demonstrated that GABA regulates SOS genes responsible for Na^+^ efflux and Na^+^/H^+^ antiporters, which are involved in sequestering excess Na^+^ into vacuoles [83]. Several other studies have also indicated that GABA under stress reduces salt ions accumulation and ROS production, activates the H^+^ ATPase and inhibits K^+^ depletion [30, 59, 61, 84, 85], which agrees with our results. GABA has also been demonstrated to interact with a variety of transporters and channels, including aluminum-activated malate transporters (ALMTs) and guard cell outward rectifying K^+^ channels (GORKs). In plants, these interactions contribute to the regulation of ion homeostasis and the enhancement of stress tolerance [86]. Thus, we propose that applying GABA to salt-stressed plants reduces the adverse effects of salinity on soybean seedlings by modulating mineral nutrient uptake and appears beneficial to plants under stressful conditions in terms of optimizing cellular metabolic processes [24, 57, 87]. Moreover, GABA influences ion membrane potential differences to enhance ion transport, thus improving plant salt tolerance [88]. As a result, we suggest that a decrease in the GABA-induced salt ion concentration is beneficial for salinity adaptation, as it is associated with a decrease in oxidative stress.

### Effect of GABA application on biochemical changes under salinity

The increased SS levels significantly enhanced proline and glycine betaine but reduced soluble sugar and soluble proteins under either stress; however, exogenous GABA enhanced their concentrations, compared to their GABA untreated peers. In plants, sugar is an important osmolyte because it regulates cell division, controls water loss, prevents chlorophyll degradation, scavenges free radicals, stabilizes membranes and proteins, and regulates gene expression [15, 89]. Proline and glycine protect the photosynthetic machinery, limit the production of excessive ROS, and stabilize enzymes, proteins, and membranes against salinity-induced damage [17, 90]. It has been reported that GABA treatment increases proline and glycine betaine under SS conditions in black pepper [91] maize [92], chufa [30], and strawberries [93], resulting in their improved tolerance. In addition, GABA-treated salinity stresses soybean seedlings exhibited improved soluble proteins, which are crucial for osmotic adjustment and can provide N when stress conditions subside [94, 95]. Consequently, our results demonstrate that GABA can enhance soybean seedlings’ ability to tolerate saline stress by enhancing the accumulation of osmolytes to mitigate the adverse effects of salt stress [24].

**Fig. 9:**
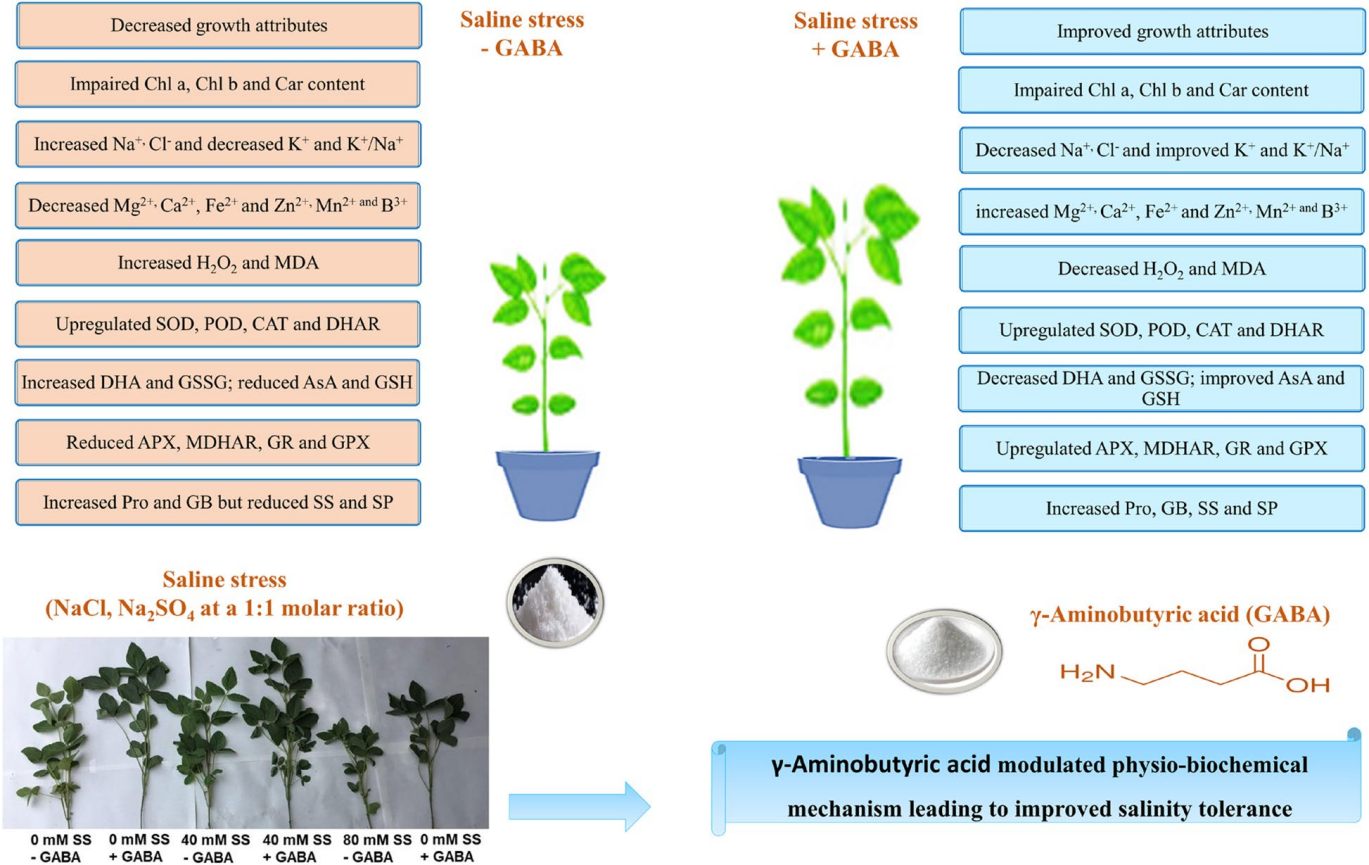
Diagram illustrating how GABA treatment affects the morpho-physiochemical attributes of soybean plants under saline stress

## Conclusion

In the present study, GABA was demonstrated to mitigate salt stress in soybean plants by modulating physio-biochemical attributes, resulting in improved growth and biomass production (Fig. 9). As a result of the increased salinity stress, soybean plants experienced adverse effects on biomass and physiological metabolism. Exogenous GABA significantly decreased soybean seedling damage caused by salt stress by (i) reducing Na^+^ and Cl^−^ accumulation, (ii) improving the accumulation of mineral ions such as Mg^2+^, Ca^2+^, Fe^2+^, Zn^2+^, and K^+^ and increasing K^+^/Na^+^ ratios, (iii) increasing osmolyte production, (iv) enhancing photosynthetic pigments (Chl a, Chl b, and Car), (v) reducing H_2_O_2_ and MDA concentrations and (vi) upregulating antioxidant enzyme activity. Hence, the findings of our study suggest that GABA application is capable of mitigating salinity-induced alterations in the morphophysiological and biochemical features of soybean plants under saline conditions. In the future, the molecular mechanisms that govern salinity tolerance mediated by GABA soybean plants should be examined further. The effects of exogenous GABA on soybean nutrition on a deeper molecular level should also be considered to resolve the low productivity of soybeans under saline conditions.

### Supplementary Information

**Supplementary Material 1.**

## Data Availability

All the data and materials are presented in the manuscript.
