# Supplementary material for: Gamma-aminobutyric acid (GABA) improves salinity stress tolerance in soybean seedlings by modulating their mineral nutrition, osmolyte contents, and ascorbate-glutathione cycle
Source: BMC Plant Biol. 2024 May 6;24:365. doi: 10.1186/s12870-024-05023-6 (PMC11071273; doi:10.1186/s12870-024-05023-6)
Supplement: Supplementary file 1 — Supplementary Material 1. [file 12870_2024_5023_MOESM1_ESM.docx]

| **Treatments** | **Radicle length (cm)** | **Plumule length (cm)** | **Seedlings fresh weight (g)** | **seedlings dry weight (g)** |
| --- | --- | --- | --- | --- |
| 0 mM | 8.7 ± 0.44 a | 7.47 ± 0.38 a | 0.70 ± 0.02 c | 0.12 ± 0.01 b |
| 40 mM | 6.10 ± 0.25 d | 3.86 ± 0.03 b | 0.41 ± 0.05 bc | 0.07 ± 0.00 b |
| 40 mM +1 mM GABA | 6.06 ± 0.51d | 4.06 ± 0.17 b | 0.43 ± 0.04 b | 0.07 ± 0.01 b |
| 40 mM +0.5 mM GABA | 6.82 ± 0.39 c | 4.52 ± 0.43 b | 0.50 ± 0.02 a | 0.08 ± 0.01 a |
| 40 mM +1.5 mM GABA | 7.74 ± 0.08 b | 6.59 ± 0.49 a | 0.69 ± 0.02 a | 0.11 ± 0.01 a |
| 40 mM +2 mM GABA | 8.23 ± 0.16 ab | 7.44 ± 0.90 a | 0.74 ± 0.06 a | 0.11 ± 0.01 a |

**Supplementary Table 1:** A preliminary growth experiments were conducted on 5 day old soybean seedlings with GABA concentrations ranging from 0.5 mM to 2 mM. We determined an optimal concentration of GABA by observing the improved growth in a saline-stressed solution containing 40 mM of GABA.

Data values were expressed as mean ± SD. According to Duncan's method, different letters exhibit significant differences (P 0.05)
